# Supplementary material for: Anti-bacterial activity of dermcidin in human platelets: suppression of methicillin-resistant Staphylococcus aureus growth
Source: Microbiol Spectr. 2025 May 27;13(7):e03273-24. doi: 10.1128/spectrum.03273-24 (PMC12210860; doi:10.1128/spectrum.03273-24)
Supplement: File S1 — Proteomic analysis techniques and methods. [file spectrum.03273-24-s0001.docx]

**Quantitative Proteomic Analysis by Tandem mass tag (TMT) technology**

**Version 2.0**

**2017.05**

**Contents**

**[1.](file:///\\\\192.168.133.198\\Share%20Folder\\公共文件夹\\yj\\整理文件夹\\ITRAQ\\英文方法说明文档\\TMT方法说明文档_english.docx#_Toc467244116)****[Experimental Instruments and Softwares](file:///\\\\192.168.133.198\\Share%20Folder\\公共文件夹\\yj\\整理文件夹\\ITRAQ\\英文方法说明文档\\TMT方法说明文档_english.docx#_Toc467244116)** [3](file:///\\\\192.168.133.198\\Share%20Folder\\公共文件夹\\yj\\整理文件夹\\ITRAQ\\英文方法说明文档\\TMT方法说明文档_english.docx#_Toc467244116)

**[2.](file:///\\\\192.168.133.198\\Share%20Folder\\公共文件夹\\yj\\整理文件夹\\ITRAQ\\英文方法说明文档\\TMT方法说明文档_english.docx#_Toc467244117)****[Materials](file:///\\\\192.168.133.198\\Share%20Folder\\公共文件夹\\yj\\整理文件夹\\ITRAQ\\英文方法说明文档\\TMT方法说明文档_english.docx#_Toc467244117)** [3](file:///\\\\192.168.133.198\\Share%20Folder\\公共文件夹\\yj\\整理文件夹\\ITRAQ\\英文方法说明文档\\TMT方法说明文档_english.docx#_Toc467244117)

**[3.](file:///\\\\192.168.133.198\\Share%20Folder\\公共文件夹\\yj\\整理文件夹\\ITRAQ\\英文方法说明文档\\TMT方法说明文档_english.docx#_Toc467244118)****[Methods](file:///\\\\192.168.133.198\\Share%20Folder\\公共文件夹\\yj\\整理文件夹\\ITRAQ\\英文方法说明文档\\TMT方法说明文档_english.docx#_Toc467244118)** [4](file:///\\\\192.168.133.198\\Share%20Folder\\公共文件夹\\yj\\整理文件夹\\ITRAQ\\英文方法说明文档\\TMT方法说明文档_english.docx#_Toc467244118)

**[3.1](file:///\\\\192.168.133.198\\Share%20Folder\\公共文件夹\\yj\\整理文件夹\\ITRAQ\\英文方法说明文档\\TMT方法说明文档_english.docx#_Toc467244119)****[Sample Preparation](file:///\\\\192.168.133.198\\Share%20Folder\\公共文件夹\\yj\\整理文件夹\\ITRAQ\\英文方法说明文档\\TMT方法说明文档_english.docx#_Toc467244119)** [4](file:///\\\\192.168.133.198\\Share%20Folder\\公共文件夹\\yj\\整理文件夹\\ITRAQ\\英文方法说明文档\\TMT方法说明文档_english.docx#_Toc467244119)

**[3.2](file:///\\\\192.168.133.198\\Share%20Folder\\公共文件夹\\yj\\整理文件夹\\ITRAQ\\英文方法说明文档\\TMT方法说明文档_english.docx#_Toc467244120)****[SDS-PAGE Separation](file:///\\\\192.168.133.198\\Share%20Folder\\公共文件夹\\yj\\整理文件夹\\ITRAQ\\英文方法说明文档\\TMT方法说明文档_english.docx#_Toc467244120)** [5](file:///\\\\192.168.133.198\\Share%20Folder\\公共文件夹\\yj\\整理文件夹\\ITRAQ\\英文方法说明文档\\TMT方法说明文档_english.docx#_Toc467244120)

**[3.3](file:///\\\\192.168.133.198\\Share%20Folder\\公共文件夹\\yj\\整理文件夹\\ITRAQ\\英文方法说明文档\\TMT方法说明文档_english.docx#_Toc467244121)****[Filter-aided sample preparation (FASP Digestion)](file:///\\\\192.168.133.198\\Share%20Folder\\公共文件夹\\yj\\整理文件夹\\ITRAQ\\英文方法说明文档\\TMT方法说明文档_english.docx#_Toc467244121)** [5](file:///\\\\192.168.133.198\\Share%20Folder\\公共文件夹\\yj\\整理文件夹\\ITRAQ\\英文方法说明文档\\TMT方法说明文档_english.docx#_Toc467244121)

**[3.4](file:///\\\\192.168.133.198\\Share%20Folder\\公共文件夹\\yj\\整理文件夹\\ITRAQ\\英文方法说明文档\\TMT方法说明文档_english.docx#_Toc467244122)****[TMT Labeling](file:///\\\\192.168.133.198\\Share%20Folder\\公共文件夹\\yj\\整理文件夹\\ITRAQ\\英文方法说明文档\\TMT方法说明文档_english.docx#_Toc467244122)** [6](file:///\\\\192.168.133.198\\Share%20Folder\\公共文件夹\\yj\\整理文件夹\\ITRAQ\\英文方法说明文档\\TMT方法说明文档_english.docx#_Toc467244122)

**[3.5](file:///\\\\192.168.133.198\\Share%20Folder\\公共文件夹\\yj\\整理文件夹\\ITRAQ\\英文方法说明文档\\TMT方法说明文档_english.docx#_Toc467244123)****[Peptide Fractionation with High pH reversed-phase](file:///\\\\192.168.133.198\\Share%20Folder\\公共文件夹\\yj\\整理文件夹\\ITRAQ\\英文方法说明文档\\TMT方法说明文档_english.docx#_Toc467244123)** [6](file:///\\\\192.168.133.198\\Share%20Folder\\公共文件夹\\yj\\整理文件夹\\ITRAQ\\英文方法说明文档\\TMT方法说明文档_english.docx#_Toc467244123)

**[3.6](file:///\\\\192.168.133.198\\Share%20Folder\\公共文件夹\\yj\\整理文件夹\\ITRAQ\\英文方法说明文档\\TMT方法说明文档_english.docx#_Toc467244132)****[Mass Spectrometry](file:///\\\\192.168.133.198\\Share%20Folder\\公共文件夹\\yj\\整理文件夹\\ITRAQ\\英文方法说明文档\\TMT方法说明文档_english.docx#_Toc467244132)** [6](file:///\\\\192.168.133.198\\Share%20Folder\\公共文件夹\\yj\\整理文件夹\\ITRAQ\\英文方法说明文档\\TMT方法说明文档_english.docx#_Toc467244132)

**[3.7](file:///\\\\192.168.133.198\\Share%20Folder\\公共文件夹\\yj\\整理文件夹\\ITRAQ\\英文方法说明文档\\TMT方法说明文档_english.docx#_Toc467244133)****[Data Analysis](file:///\\\\192.168.133.198\\Share%20Folder\\公共文件夹\\yj\\整理文件夹\\ITRAQ\\英文方法说明文档\\TMT方法说明文档_english.docx#_Toc467244133)** [7](file:///\\\\192.168.133.198\\Share%20Folder\\公共文件夹\\yj\\整理文件夹\\ITRAQ\\英文方法说明文档\\TMT方法说明文档_english.docx#_Toc467244133)

**[4.](file:///\\\\192.168.133.198\\Share%20Folder\\公共文件夹\\yj\\整理文件夹\\ITRAQ\\英文方法说明文档\\TMT方法说明文档_english.docx#_Toc467244134)****[Bioinformatic Analysis](file:///\\\\192.168.133.198\\Share%20Folder\\公共文件夹\\yj\\整理文件夹\\ITRAQ\\英文方法说明文档\\TMT方法说明文档_english.docx#_Toc467244134)** [8](file:///\\\\192.168.133.198\\Share%20Folder\\公共文件夹\\yj\\整理文件夹\\ITRAQ\\英文方法说明文档\\TMT方法说明文档_english.docx#_Toc467244134)

**[4.1](file:///\\\\192.168.133.198\\Share%20Folder\\公共文件夹\\yj\\整理文件夹\\ITRAQ\\英文方法说明文档\\TMT方法说明文档_english.docx#_Toc467244135)****[Gene Ontology (GO) Annotation](file:///\\\\192.168.133.198\\Share%20Folder\\公共文件夹\\yj\\整理文件夹\\ITRAQ\\英文方法说明文档\\TMT方法说明文档_english.docx#_Toc467244135)** [8](file:///\\\\192.168.133.198\\Share%20Folder\\公共文件夹\\yj\\整理文件夹\\ITRAQ\\英文方法说明文档\\TMT方法说明文档_english.docx#_Toc467244135)

**[4.2](file:///\\\\192.168.133.198\\Share%20Folder\\公共文件夹\\yj\\整理文件夹\\ITRAQ\\英文方法说明文档\\TMT方法说明文档_english.docx#_Toc467244137)****[KEGG Pathway Annotation](file:///\\\\192.168.133.198\\Share%20Folder\\公共文件夹\\yj\\整理文件夹\\ITRAQ\\英文方法说明文档\\TMT方法说明文档_english.docx#_Toc467244137)** [8](file:///\\\\192.168.133.198\\Share%20Folder\\公共文件夹\\yj\\整理文件夹\\ITRAQ\\英文方法说明文档\\TMT方法说明文档_english.docx#_Toc467244137)

**[4.3](file:///\\\\192.168.133.198\\Share%20Folder\\公共文件夹\\yj\\整理文件夹\\ITRAQ\\英文方法说明文档\\TMT方法说明文档_english.docx#_Toc467244138)****[Functional Enrichment analysis](file:///\\\\192.168.133.198\\Share%20Folder\\公共文件夹\\yj\\整理文件夹\\ITRAQ\\英文方法说明文档\\TMT方法说明文档_english.docx#_Toc467244138)** [8](file:///\\\\192.168.133.198\\Share%20Folder\\公共文件夹\\yj\\整理文件夹\\ITRAQ\\英文方法说明文档\\TMT方法说明文档_english.docx#_Toc467244138)

**[4.4](file:///\\\\192.168.133.198\\Share%20Folder\\公共文件夹\\yj\\整理文件夹\\ITRAQ\\英文方法说明文档\\TMT方法说明文档_english.docx#_Toc467244139)****[Hierarchical Clustering](file:///\\\\192.168.133.198\\Share%20Folder\\公共文件夹\\yj\\整理文件夹\\ITRAQ\\英文方法说明文档\\TMT方法说明文档_english.docx#_Toc467244139)** [9](file:///\\\\192.168.133.198\\Share%20Folder\\公共文件夹\\yj\\整理文件夹\\ITRAQ\\英文方法说明文档\\TMT方法说明文档_english.docx#_Toc467244139)

**[4.5](file:///\\\\192.168.133.198\\Share%20Folder\\公共文件夹\\yj\\整理文件夹\\ITRAQ\\英文方法说明文档\\TMT方法说明文档_english.docx#_Toc467244140)****[Protein-Protein Interact Network（PPI）](file:///\\\\192.168.133.198\\Share%20Folder\\公共文件夹\\yj\\整理文件夹\\ITRAQ\\英文方法说明文档\\TMT方法说明文档_english.docx#_Toc467244140)** [9](file:///\\\\192.168.133.198\\Share%20Folder\\公共文件夹\\yj\\整理文件夹\\ITRAQ\\英文方法说明文档\\TMT方法说明文档_english.docx#_Toc467244140)

**[5.](file:///\\\\192.168.133.198\\Share%20Folder\\公共文件夹\\yj\\整理文件夹\\ITRAQ\\英文方法说明文档\\TMT方法说明文档_english.docx#_Toc467244141)****[Reference](file:///\\\\192.168.133.198\\Share%20Folder\\公共文件夹\\yj\\整理文件夹\\ITRAQ\\英文方法说明文档\\TMT方法说明文档_english.docx#_Toc467244141)** [9](file:///\\\\192.168.133.198\\Share%20Folder\\公共文件夹\\yj\\整理文件夹\\ITRAQ\\英文方法说明文档\\TMT方法说明文档_english.docx#_Toc467244141)

1. **Experimental Instruments and Software**

| Experimental Instruments： | |
| --- | --- |
| Easy nLC Liquid Chromatograph (Thermo Scientific) | Q Exactive Mass Spectrometer（Thermo Scientific） |
| Nanodrop 2000c (Thermo Scientific) | Multiskcan FC Microplate Photometer（Thermo Scientific） |
| Centrifuges（Eppendorf 5430R） | Concetrator plus/Vacufuge（Eppendorf Concentrator Plus） |
| Electrophoresis（GE Healthcare EPS601） | MP Fastprep-24 Automated Homogenizer（MP Biomedicals) |
| Ultrasonic Liquid Processors（Scientz JY92-II，Ningbo） | Electric Thermostatic Incubator（Jinghong GNP-9080, Shanghai） |
| Vortex（QiTe QT-1，Shanghai） | Electronic balance（METLER TOLED AL104） |
| Software： | |
| ProteomeDiscoverer 1.4(Thermo Scientific) | MASCOT 2.2 (Matrix Science) |
| Perseus 1.3 ([Max Planck Institute of Biochemistry](https://en.wikipedia.org/wiki/Max_Planck_Institute_of_Biochemistry) in [Martinsried](https://en.wikipedia.org/wiki/Martinsried), [Germany](https://en.wikipedia.org/wiki/Germany)) | \ |

1. **Materials**

| Glycerol（G0854, Sangon / 500ml） | Bromophenol Blue（161‐0404, Sangon） |
| --- | --- |
| SDS (161‐0302, Bio‐Rad) | Urea (161‐0731, Bio‐Rad) |
| Tris (A6141 ,Sigma) | DTT (161‐0404, Bio‐Rad) |
| Iodoaceamide ( IAA , 163‐2109, Bio‐Rad) | KH_2_PO_4_（10017618, Sinopharm） |
| KCl (10016318, Sinopharm) | HCl（10011018, Sinopharm） |
| BCA Protein Assay Kit（P0012, Beyotime） | BSA（A0332-25G, Sangon） |
| 84868 Pierce™ High pH Reversed-Phase Peptide Fractionation Kit (12 reactions) | Trypsin (317107, Promega) |
| Lysing matrix A（MP 6910-050） | 1/4 ceramic sphere （MP 6540-034） |
| Formic Acid（FA, 06450, Fluka） | Trifluoroacetic Acid（TFA , T6508, Sigma） |
| Acetonirile (ACN，I592230123, Merck) | 10kD Ultrafiltration Tube (Sartorius) |
| C18 Cartridge (66872‐U, Sigma) | Multiple Affinity Removal LC Column – Human 14 / Mouse 3 (Agilent) |
| TMT 6/10 plex Isobaric Label Reagent (Thermo) | |
| C18 Trap Column：Thermo Scientific Acclaim PepMap100, 100μm*2cm, nanoViper C18, 3μm, 100 Å | |
| C18 Analytical Column：Thermo scientific EASY column, 10cm, ID75μ m, 3μm, C18-A2 | |
| 5X Loadig Buffer：10% SDS，0.5% Bromophenol Blue，50% Glycerol，500mM DTT，250mM Tris-HCl, pH6.8 | |
| SDT Lysis Buffer：4%SDS，100mM Tris-HCl，1mM DTT，pH7.6 | |
| UA buffer：8M urea，150mM Tris-HCl，pH 8.0 | |
| HPLC Buffer A：0.1%FA | |
| HPLC Buffer B：0.1% FA, 84% ACN | |

1. **Methods**
   1. **Sample Preparation**

**The method was determined by the project proposal or preliminary experiment report.**

1. **TCA/Acetone Precipitation and SDT Lysis^[1]^：**

**Application: Plant tissues (roots, stems ,lea****ves, etc）, hard tissues（skin, cartilage, hair, etc）, fungi.**

The amount samples were frozen in liquid nitrogen and ground with a pestle and mortar. 5 times volume of TCA/acetone (1:9) was added to the powder and mixed by vortex. The mixture was placed at -20℃ for 4h, and centrifuged at 6000g for 40 min at 4℃. The supernatant was discarded. The pre-cooling acetone was added and washed for three times. The precipitation was air dried. 30 times volume of SDT buffer was added to 20-30 mg powder, mixed and boiled for 5 min. The lysate was sonicated and then boiled for 15 min. After centrifuged at 14000g for 40 min, the supernatant was filtered with 0.22 µm filters. The filtrate was quantified with the BCA Protein Assay Kit (Bio-Rad, USA). The sample was stored at -80 °C.

1. **Homogenate and SDT Lysis^[2]^：**

**Application: Tender tissues (brain, liver, muscle, etc), mollusk, microorganism, etc.**

SDT buffer was added to the sample, and transferred to 2 ml tubes with amount quartz sand (another 1/4 inch ceramic bead MP 6540-424 for tissue samples). The lysate was homogenized by MP homogenizer (24×2, 6.0M/S, 60s, twice). The homogenate was sonicated and then boiled for 15 min. After centrifuged at 14000g for 40 min, the supernatant was filtered with 0.22 µm filters. The filtrate was quantified with the BCA Protein Assay Kit (Bio-Rad, USA). The sample was stored at -80 °C.

1. **SDT Lysis^[3]^：**

**Application：Cell, protein powder, body fluid, concentrated fermentation broth, cell secretion, etc.**

SDT buffer was added to the sample. The lysate was sonicated (this step can be skipped for protein solution) and then boiled for 15 min. After centrifuged at 14000g for 40 min, the supernatant was quantified with the BCA Protein Assay Kit (Bio-Rad, USA). The sample was stored at -80 °C.

1. **Immunoaffinity Depletion of Serum High-Abundance Proteins**

**Application： human, mouse or rat serum**

Serum pools were depleted of most abundant proteins using Agilent Human 14 / Mouse 3 Multiple Affinity Removal System Column following the manufacturer’s protocol **^[4-6]^** (Agilent Technologies). The Human 14 column was applied for human, and Mouse 3 column was applied for mouse and rat. The 10 kDa ultrafiltration tube (Sartorius) was used for desalination and concentration of low-abundance components. One volume of SDT buffer was added, boiled for 15min and centrifuged at 14000g for 20 min. The supernatant was quantified with the BCA Protein Assay Kit (Bio-Rad, USA). The sample was stored at -80 °C.

- 1. **SDS-PAGE Separation**

20 µg of proteins for each sample were mixed with 5X loading buffer respectively and boiled for 5 min. The proteins were separated on 12.5% SDS-PAGE gel (constant current 14 mA, 90 min). Protein bands were visualized by Coomassie Blue R-250 staining.

- 1. **Filter-aided sample preparation (FASP Digestion)^[3]^**

200 μg of proteins for each sample were incorporated into 30 μl SDT buffer (4% SDS, 100 mM DTT, 150 mM Tris-HCl pH 8.0). The detergent, DTT and other low-molecular-weight components were removed using UA buffer (8 M Urea, 150 mM Tris-HCl pH 8.0) by repeated ultrafiltration (Microcon units, 10 kD). Then 100 μl iodoacetamide (100 mM IAA in UA buffer) was added to block reduced cysteine residues and the samples were incubated for 30 min in darkness. The filters were washed with 100 μl UA buffer three times and then 100 μl 100 mM TEAB buffer twice. Finally, the protein suspensions were digested with 4 μg trypsin (Promega) in 40 μl TEAB buffer overnight at 37 °C, and the resulting peptides were collected as a filtrate. The peptide content was estimated by UV light spectral density at 280 nm using an extinctions coefficient of 1.1 of 0.1% (g/l) solution that was calculated on the basis of the frequency of tryptophan and tyrosine in vertebrate proteins.

- 1. **TMT Labeling**

100 μg peptide mixture of each sample was labeled using TMT reagent ^[7]^ according to the manufacturer’s instructions (Thermo Fisher Scientific).

- 1. **Peptide Fractionation with High pH reversed-phase**

Pierce high pH reversed-phase fractionation kit^[8]^(Thermo scientific) was used to fractionate TMT-labeled digest samples into 10 (or 15 determined by project proposal) fractions by an increasing acetonitrile step-gradient elution according to instructions.

- 1. **Mass Spectrometry**
     1. **HPLC**

Each fraction was injected for nanoLC-MS/MS analysis. The peptide mixture was loaded onto a reverse phase trap column（Thermo Scientific Acclaim PepMap100, 100μm*2cm, nanoViper C18）connected to the C18-reversed phase analytical column (Thermo Scientific Easy Column, 10 cm long, 75 μm inner diameter, 3μm resin) in buffer A (0.1% Formic acid) and separated with a linear gradient of buffer B (84% acetonitrile and 0.1% Formic acid) at a flow rate of 300 nl/min controlled by IntelliFlow technology. The linear gradient was determined by the project proposal:

1. 1 hour gradient：0-50% buffer B for 50 min, 50-100% buffer B for 5 min, hold in 100% buffer B for 5 min.
2. 1.5 hours gradient：0-55% buffer B for 80 min, 55-100% buffer B for 5 min, hold in 100% buffer B for 5 min.
   - 1. **LC-MS/MS Analysis**

LC-MS/MS analysis was performed on a Q Exactive mass spectrometer (Thermo Scientific) that was coupled to Easy nLC (Proxeon Biosystems, now Thermo Fisher Scientific) for 60/90 min (determined by project proposal). The mass spectrometer was operated in positive ion mode. MS data was acquired using a data-dependent top10 method dynamically choosing the most abundant precursor ions from the survey scan (300–1800 m/z) for HCD fragmentation. Automatic gain control (AGC) target was set to 3e6, and maximum inject time to 10 ms. Dynamic exclusion duration was 40.0 s. Survey scans were acquired at a resolution of 70,000 at m/z 200 and resolution for HCD spectra was set to 17500 at m/z 200 (TMT 6plex), 35000 at m/z 200 (TMT 10plex), and isolation width was 2 m/z.. Normalized collision energy was 30 eV and the underfill ratio, which specifies the minimum percentage of the target value likely to be reached at maximum fill time, was defined as 0.1%. The instrument was run with peptide recognition mode enabled.

- 1. **Data Analysis**

MS/MS spectra were searched using MASCOT engine (Matrix Science, London, UK; version 2.2) embedded into Proteome Discoverer 1.4 ^[9]^. The following parameters were set.

| **Item** | **Value** |
| --- | --- |
| **Enzyme** | Trypsin |
| **Max Missed Cleavages** | 2 |
| **Fixed modifications** | Carbamidomethyl (C),  TMT6/10plex (N-term), TMT6/10plex (K) |
| **Variable modifications** | Oxidation (M) |
| **Peptide Mass Tolerance** | ± 20 ppm |
| **Fragment Mass Tolerance** | 0.1Da |
| **Database** | See the project report |
| **Database pattern** | Decoy |
| **Peptide FDR** | ≤0.01 |
| **Protein Quantification** | The protein ratios are calculated as the median of only unique peptides of the protein |
| **Experimental Bias** | Normalizes all peptide ratios by the median protein ratio. The median protein ratio should be 1 after the normalization. |

1. **Bioinformatic Analysis**
   1. **Gene Ontology (GO) Annotation**

The protein sequences of differentially expressed proteins were in batches retrieved from UniProtKB database (Release 2016_10) in FASTA format. The retrieved sequences were locally searched against SwissProt database (mouse) using the NCBI BLAST**+** client software (ncbi-blast-2.2.28+-win32.exe) to find homologue sequences from which the functional annotation can be transferred to the studied sequences. In this work, the top 10 blast hits with E-value less than 1*e*-3 for each query sequence were retrieved and loaded into Blast2GO^10^ (Version 3.3.5) for GO mapping and annotation. In this work, an annotation configuration with an E-value filter of 1*e*-6, default gradual EC weights, a GO weight of 5, and an annotation cutoff of 75 were chosen. Un-annotated sequences were then re-annotated with more permissive parameters. The sequences without BLAST hits and un-annotated sequences were then selected to go through an InterProScan^11^ against EBI databases to retrieve functional annotations of protein motifs and merge the InterProScan GO terms to the annotation set. The GO annotation results were plotted by R scripts.

- 1. **KEGG Pathway Annotation**

The FASTA protein sequences of differentially changed proteins were blasted against the online Kyoto Encyclopedia of Genes and Genomes (KEGG) database (http://geneontology.org/) to retrieve their KOs and were subsequently mapped to pathways in KEGG^12^. The corresponding KEGG pathways were extracted.

- 1. **Functional Enrichment analysis**

To further explore the impact of differentially expressed protein in cell physiological process and discover internal relations between differentially expressed proteins, enrichment analysis was performed. GO enrichment on three ontologies (biological process, molecular function, and cellular component) and KEGG pathway enrichment analyses were applied based on the Fisher’ exact test, considering the whole quantified protein annotations as background dataset. Benjamini-Hochberg correction for multiple testing was further applied to adjust derived p-values. And only functional categories and pathways with p-values under a threshold of 0.05 were considered as significant.

- 1. **Hierarchical Clustering**

The studied protein relative expression data was used to performing hierarchical clusteringanalysis.Forthispurpose,Cluster3.0(http://bonsai.hgc.jp/~mdehoon/software/cluster/software.htm) and the Java Treeview software (http://jtreeview.sourceforge.net) were used. Euclidean distance algorithm for similarity measure and average linkage clustering algorithm (clustering uses the centroids of the observations) for clustering were selected when performing hierarchical clustering. Heatmap is often presented as a visual aid in addition to the dendrogram.

- 1. **Protein-Protein Interact Network（PPI）**

The protein–protein interaction information of the studied proteins was retrieved from IntAct molecular interaction database (http://www.ebi.ac.uk/intact/) by their gene symbols or STRING software (<http://string-db.org/>). The results were downloaded in the XGMML format and imported into Cytoscape software (http://www.cytoscape.org/,version 3.2.1) to visualize and further analyze functional protein-protein interaction networks. Furthermore, the degree of each protein was calculated to evaluate the importance of the protein in the PPI network.

1. **Reference**
2. [Plant Proteomics: Methods and Protocols](http://www.smarter.com.cn/redir.php?bt=b2ZmZXI%3D&ch=1011&oi=28025221&mc=1&dp=1&pr=0&rr=0&sb=&cb=). Hervé Thiellement, Michel Zivy, Catherine Damerval, and Valerie Mechin, 2007. METHODS IN MOLECULAR BIOLOGY 355.
3. Proteomic analysis of solid pseudopapillary tumor of the pancreas reveals dysfunction of the endoplasmic reticulum protein processing pathway. Zhu Y et al. Mol Cell Proteomics.2014. 13(10):2593-603.
4. Universal sample preparation method for proteome analysis. Wisniewski, J. R., A. Zougman, et al. Nat Methods.2009. 6(5): 359-362.
5. Agilent Human 14 Multiple Affinity Removal System Columns for the Fractionation of High- Abundant Proteins from Human Proteomic Samples. Agilent Technologies, Inc. 2007.
6. Agilent Multiple Affinity Removal Columns – for Mouse Serum Proteins. Agilent Technologies, Inc. 2005.
7. Immunodepletion of High-Abundant Proteins from Rat Serum with the Agilent Multiple Affinity Removal System for Mouse. Agilent Technologies, Inc. 2004.
8. TMT Mass Tagging Kits and Reagents. Thermo Fisher Scientific.
9. User Guide: Pierce High pH Reversed-Phase Peptide Fractionation Kit
10. Proteome Discoverer Version 1.4. Thermo Fisher Scientific Inc. 2012.
11. High-throughput functional annotation and data mining with the Blast2GO suite. Götz S, García-Gómez JM, et al. Nucleic Acids Res. 2008; 36(10): 3420-35.
12. InterProScan: protein domains identifier. Quevillon E, Silventoinen V, et al. Nucleic Acids Res. 2005; 33(Web Server issue): W116-20.
13. KAAS: an automatic genome annotation and pathway reconstruction server. Moriya Y, Itoh M, Okuda S, Yoshizawa AC, Kanehisa M. Nucleic Acids Res. 2007 Jul;35(Web Server issue):W182-5.
