# Supplementary material for: Anti-bacterial activity of dermcidin in human platelets: suppression of methicillin-resistant Staphylococcus aureus growth
Source: Microbiol Spectr. 2025 May 27;13(7):e03273-24. doi: 10.1128/spectrum.03273-24 (PMC12210860; doi:10.1128/spectrum.03273-24)
Supplement: Legends — for Files S1 and S2. [file spectrum.03273-24-s0003.docx]

**Supplementary material** **legends**

**Supplementary material 1. Proteomic analysis techniques and methods.** The supernatant proteins of platelets co-cultured with or without MRSA for 10 h were collected and sent to Shanghai Zhongke New Life Biotechnology Co. for quantitative proteomic analysis by tandem mass tag (TMT) technology. Supplementary Material 1 presents the detailed techniques and methods for proteomics experimental analysis.

**Supplementary material 2. The list of proteins identified by proteomics analysis.** In total, 2304 proteins with unique peptides or polypeptide segments were identified by proteomics analysis. According to the screening standard, multiple changes were greater than 1.4 (up-regulation greater than 1.4, or downregulation less than 0.714) and the *P*-value was less than 0.05; a total of 581 differentially expressed proteins (DEPs) were screened. Compared to the PLT group, 295 proteins were significantly downregulated in the PLT-MRSA group (P-M/P vertical column marked with blue), whereas 286 proteins were significantly upregulated (P-M/P vertical column marked with red). Line 547 shows the dermcidin (DCD) protein (Accession: P81605).
